# Reappraisal of the clinical and anatomic characteristics of idiopathic outflow tract ventricular arrhythmias with an R wave pattern break in precordial lead: A multi-center study

**DOI:** 10.1016/j.ijcha.2025.101664

**Published:** 2025-03-25

**Authors:** Muhammad Rafdi Amadis, Satoshi Higa, Chin-Yu Lin, Yuen Hoong Phang, Chia-Hsin Chiang, Jose Antonio Lopez Bautista, Yenn-Jiang Lin, Shih-Lin Chang, Li-Wei Lo, Yu-Feng Hu, Fa-Po Chung, Ting-Yung Chang, Ling Kuo, Cheng-I Wu, Chih-Min Liu, Shin-Huei Liu, Ming-Jen Kuo, Thien-Chuong Nguyen-Khac, Guan-Yi Li, Yu-Shan Huang, Shih-Ann Chen

**Affiliations:** a Division of Cardiology, Department of Medicine, Taipei Veterans General Hospital, Taipei, Taiwan; b Cardiovascular Center, Taichung Veterans General Hospital, Taichung, Taiwan; c Cardiac Electrophysiology and Pacing Laboratory, Division of Cardiovascular Medicine, Makiminato Central Hospital, Okinawa, Japan; d Institute of Clinical Medicine, National Yang-Ming Chiao-Tung University, Taipei, Taiwan; e Department of Biomedical Sciences and Engineering, National Central University, Taoyuan, Taiwan; f National Chung Hsing University, Taichung, Taiwan; g Department of Medicine, China Medical University College of Medicine, Taichung, Taiwan

**Keywords:** Ventricular arrhythmia, Pattern break, Right ventricular outflow tract, Ablation, Commissure of the left coronary cusp and right coronary cusp

## Abstract

**Background:**

Idiopathic outflow tract ventricular arrhythmia (OT-VA) with a pattern break (PB) in the precordial leads is challenging to treat and is associated with low success rates.

**Objective:**

Describe the anatomic characteristics and outcomes of patients with PB idiopathic OT-VA underwent catheter ablation.

**Methods:**

We retrospectively reviewed the electronic medical records of idiopathic OT-VA patients underwent catheter ablation at Taipei Veterans General Hospital, Taiwan, and Makiminato Central Hospital, Japan. Patients with a documented left bundle branch block (LBBB) pattern and inferior axis VA QRS morphology with PB were included. Clinical data, VA morphology, electrophysiological parameters, and anatomic characteristics were analyzed.

**Results:**

A total of 66 patients (6.7 %) had a PB in V2 (N = 60) or V3 (N = 6) were identified. The acute and long-term success rates (after a median follow-up of 37 months) were 92.4 % and 78.8 %, respectively, higher than previously reported. The successful ablation sites were mainly the right ventricular outflow tract (RVOT, 81.8 % [54/66]). The earliest activation site in the LVOT was mainly the left coronary cusp (LCC)/right coronary cusp (RCC) commissure (95.7 %, 22/23). A long anatomic distance from the LCC/RCC commissure to the RVOT and diabetes mellitus (DM) independently predicted acute procedural failure or recurrence.

**Conclusion:**

OT-VA with PB in the Asian population may not have worse clinical outcomes than previously reported in Western countries. The most common site of acute success for ablation was the RVOT. A long anatomic distance from the LCC/RCC commissure to the RVOT and DM diagnosis was associated with an unsatisfied ablation outcome.

## 1 Background

Idiopathic ventricular arrhythmia (VA) originating from the outflow tract (OT) represents a common cardiac arrhythmia in our clinical setting. Catheter ablation has become a standard therapy, especially for idiopathic VA originating from the right ventricular outflow tract (RVOT), as it confers an excellent clinical outcome compared with pharmacological therapy [1], [2]. Recent consensus also recommended catheter ablation for idiopathic premature ventricular contraction (PVC) or ventricular tachycardia (VT) originating from the OT, especially for patients with frequent and symptomatic patients, or if drug therapy is not effective, not tolerated, or not desired [3].

However, a previous report described a poor acute and late success rate in a subset of patients presenting with a pattern break (PB) in lead V2, characterized by the R wave in V2 being less positive than in V1 and V3. This is likely because the VA originated from the anterior interventricular sulcus, anatomically opposite to lead V2. The close proximity of the arrhythmia origin to the left anterior descending (LAD) coronary artery makes ablation difficult [4], [5].

In this study, we described the incidence, clinical characteristics, anatomic information in the successful ablation site, and outcome of catheter ablation in patients with VA characterized by an R wave pattern break in the precordial lead in an Asian population.

## 2 Methods

### 2.1 Study population

We included all consecutive patients who underwent catheter ablation of idiopathic VA at Taipei Veterans General Hospital (Taiwan) between October 2018 and 30 September 2023 and from Makiminato Central Hospital, Japan, between June 2016 and May 2023. We retrospectively reviewed the medical record regarding symptoms, comorbidity, and other clinical characteristics. All patients underwent transthoracic echocardiography (TTE) and/or cardiac magnetic resonance (CMR) to rule out the presence of structural heart disease. This study was approved by the ethics committee at each institution. Informed consent was waived because of the retrospective nature of the study and the analysis used anonymous clinical data.

### 2.2 Electrocardiographic analysis

**Fig. 1:**
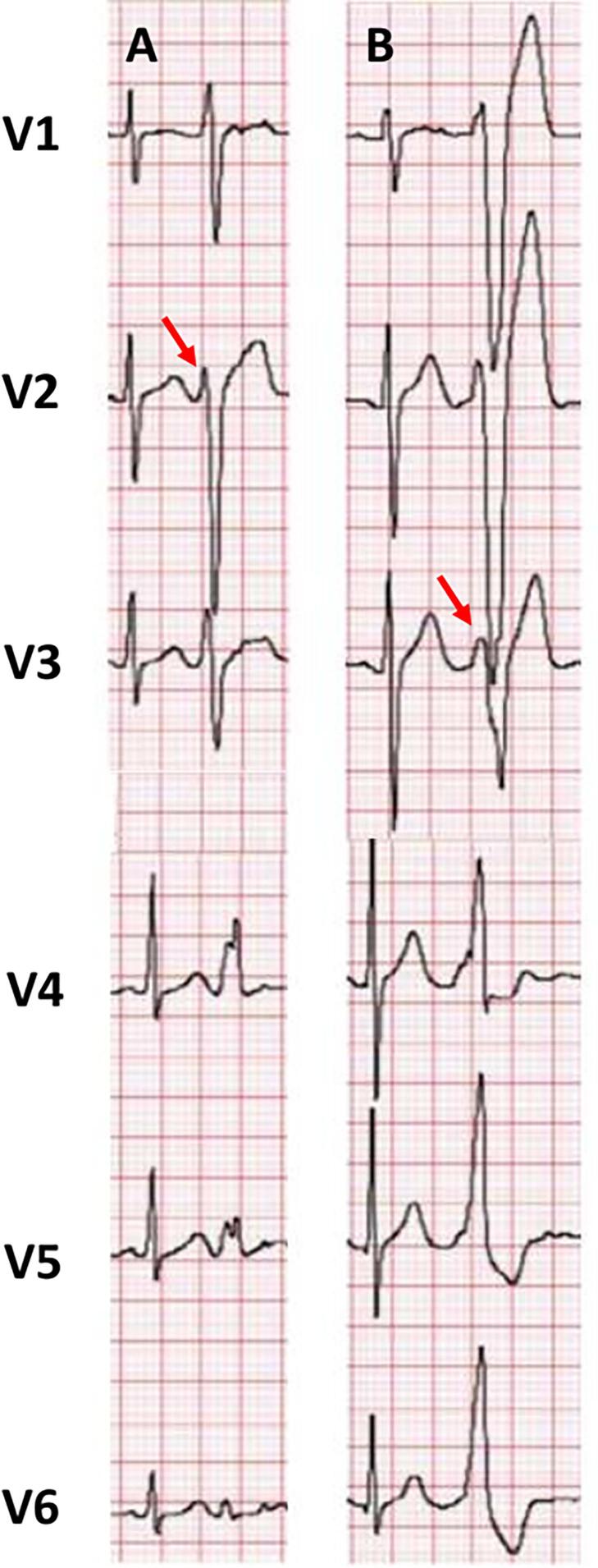
Comparison of the precordial lead between PBV2 (Fig. 1A) and PBV3 (Fig. 1B). Abbreviation: PBV2**,** pattern break at V2; PBV3, pattern break at V3.

All patients with documented break patterns in precordial leads on standard 12-lead ECG were included. We defined pattern break at V2 (PBV2) as the net QRS amplitude of V2 being less positive or having a smaller R wave compared to V1 and V3 (Fig. 1**A**) [4]. We also included patients with pattern break at V3 (PBV3), defined as the net QRS amplitude of V3 being less positive or having a smaller R wave compared to V2 and V4 (Fig. 1**B**).

Detailed ECG analysis was performed offline on the Labsystem™ Pro EP Recording System (Boston Scientific), with the recordings displayed at a speed of 100 and 200 mm/s. ECG tracings were analyzed according to previously reported measurements applied to the outflow tract region. In particular, the following ECG features were assessed: 1) QRS duration; 2) ratio between R wave in lead II / lead III; 3) Q wave amplitude ratio in leads aVR / aVL; 4) absence of a QS complex in lead I; 5) precordial transition in left bundle branch block morphology. Three QRS complexes were measured in each patient to confirm the reproducibility, and the averaged values were used. In order to formally assess the degree of inter-observer variability in measurements, two observers independently measured these ECG parameters from randomly selected patients within the study cohort. Concordance in measurements was defined as less than 5 ms variability for timing and less than 0.2 mV for amplitude ratio. The observers were blinded to the results of the ablation procedure.

### 2.3 Electrophysiology study, mapping, and catheter ablation

All patients had failed treatment with at least one antiarrhythmic drug. Antiarrhythmic drugs were discontinued for at least five half-lives before radiofrequency catheter ablation (RFCA). All patients signed a written informed consent form according to the hospital’s institutional guidelines. The electrophysiology study and ablation were performed under local anesthesia or deep sedation for the patient’s comfort at the operator’s discretion. Standard twelve-lead surface ECG was applied to all patients during the procedure. Electroanatomic mapping was performed using either the Carto 3 mapping system (Biosense Webster, Inc) or EnSite Precision (Abbott, Inc). Mapping was performed using a multi-electrode mapping catheter and/or 3.5-mm open-irrigated ablation catheter (Thermocool, Biosense Webster Inc; FlexAbility, Abbott). Activation mapping was performed to identify the site with the best local activation time (LAT). LAT was defined as the interval from the local potential onset in mapping catheter compared with the earliest onset of QRS in ECG. When clinically relevant VA occurs spontaneously, a mapping catheter was positioned in the RVOT to map the site with the earliest LAT. We mapped the left ventricular outflow tract (LVOT) and/or great cardiac vein (GCV) and anterior interventricular vein (AIVV) if we could not find a site with a good LAT and pace mapping in RVOT.

When the clinical VA was absent or infrequent, we administered isoproterenol up to 4 µg/min with or without ventricular burst pacing from RV apex and basal area. Once the clinical VA was present, but still a very limited number, we mainly relied on pace mapping with the output of ≤10 mA and 2 ms pulse width. Good pace mapping was defined as more than 90 % similarity with clinical spontaneous VA. Both activation mapping and pace mapping were used to predict the site of origin, if possible. Radiofrequency energy was delivered at the site with the earliest LAT and/or good pace mapping. Radiofrequency energy was delivered initially at the earliest activation site via an irrigated tip catheter with a maximal power of 30 – 40 Watts for the endocardium, 25 – 35 Watts for the cusp, and 20 – 30 Watts for the coronary sinus, respectively. The radiofrequency application was continued and carefully titrated for a minimum of 120 s of the VA immediately (<10 s) during ablation. Once the VA was suppressed after ablation for more than 10 s, additional energy was applied with a minimal duration of 180 s and a maximum of 300 s. If the VA persisted after ablation, the mapping protocol was repeated to identify the earliest activation site. Once ablation was performed at the LVOT or coronary sinus, the location of the left/right coronary artery was confirmed by coronary angiography to prevent any accidental coronary vessel injury. We defined VA origin as the final successful site that suppressed the VA. When we could not suppress the VA, the VA origin was assumed to be where we get the earliest LAT and best pace map.

Acute procedural success was defined by the complete elimination of any spontaneous or inducible VAs under an isoproterenol infusion (4 μg/min), following the same induction protocol in all patients. The observation time was 30 min after the final ablation. The locations of the VAs were identified by three-dimensional navigation system mapping and fluoroscopy.

### 2.4 Distance between left coronary cusp (LCC)/right coronary cusp (RCC) commissure and RVOT

**Fig. 2:**
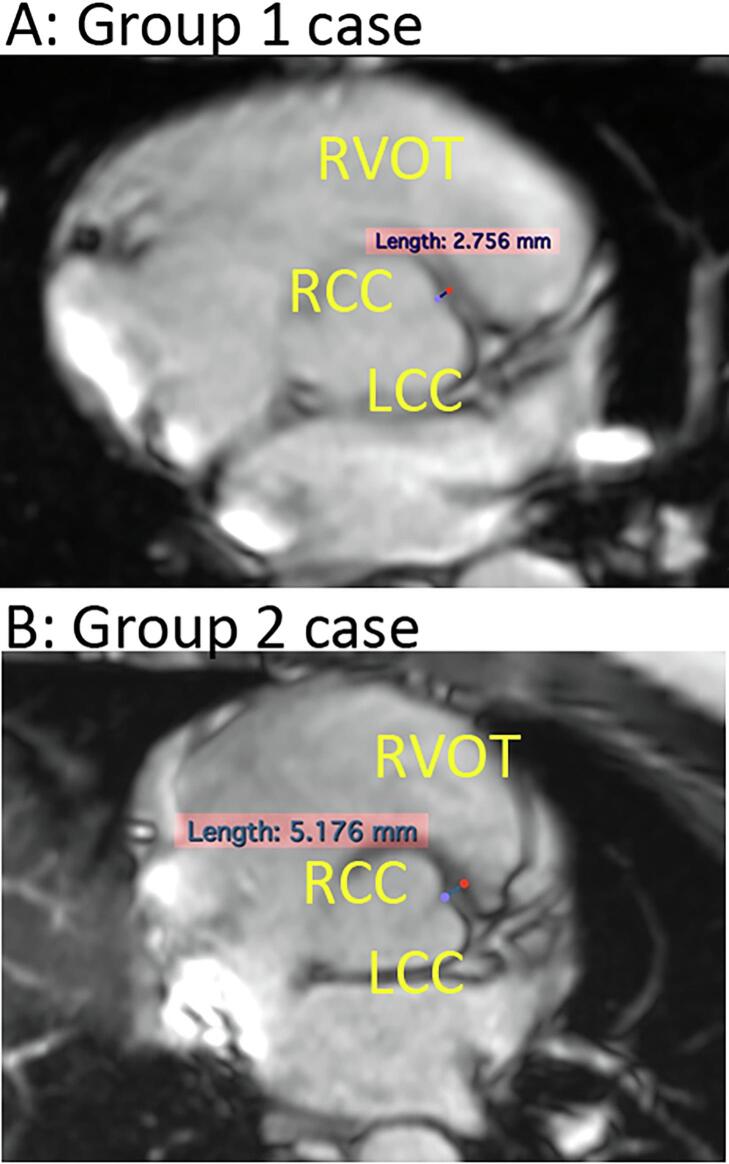
Example of measurement of distance from LCC/RCC commissure (blue point) to RVOT (red point) by using the Horos software. We measured the shortest distance between the LCC/RCC commissure and the RVOT in the plane horizontal to the aortic cusps. A: example of a patient from group 1; B: example of a patient from group 2. Abbreviation: LCC, left coronary cusp; RCC, right coronary cusp; RVOT, right ventricular outflow tract.

The distance between the LCC/RCC commissure and RVOT septum was measured using the Horos software. Horos is an open-source version of OsiriX (Pixmeof, Switzerland), which allows the processing of three-dimensional datasets [6]. The imaging of CMR was exported for analysis. We measured the shortest anatomical distance between the LCC/RCC commissure and the RVOT in a plane parallel to the alignment of the LCC and RCC. The measurement was performed by two cardiovascular technicians blinded to the patient’s information. (Fig. 2).

### 2.5 Follow up

Patients were followed up in the cardiology outpatient clinic with twelve-lead ECGs and 24-hour Holter after RFCA every three months for the first half-year and then six months afterwards. Patients who could not come for outpatient follow-up in our institution were advised to visit our affiliated institutions to complete follow-up screening. The medical reports were obtained from these affiliated institutions.

Recurrence of VA was defined as the presence of VT or PVC with a burden of >1000 per day on 24-hour Holter monitoring with a similar morphology to initial VA. Patients with successful ablation without recurrence for at least 1 year were classified as Group 1. The others were classified as Group 2.

### 2.6 Statistical analysis

All statistical analyses were performed with statistical software SPSS version 25 (IBM, Armonk, New York, USA). Continuous variables were expressed as mean ± SD or median with interquartile range (IQR) as appropriate. A comparison between continuous variables between groups was analyzed using an independent T-test. Categorical variables are expressed as numbers and percentages. A comparison between categorical variables was analyzed using the Chi-square or Fischer exact test as appropriate. P < 0.05 was considered statistically significant. Variables selected for odds ratio evaluation were age, gender, and other parameters with *P* < 0.1 in the prior comparison. Variables selected for multivariate analysis were parameters with *P* < 0.1 in the univariate model. The odds ratio was significant when the 95 % confidence interval (CI) exceeded 1.0 and the *P* value was <0.05. A *P* value < 0.05 was considered statistically significant.

## 3 Results

Nine hundred eighty-four patients underwent ablation for idiopathic VA from two institutions during the specified period. Of these patients, 66 (6.7 %) presented with pattern breaks in precordial leads. Among these, 60 patients had a PBV2 (90.9 %), corresponding with 6.1 % of all patients undergoing idiopathic VA ablation. The remaining six patients (9.1 %) had a PBV3, corresponding with 0.6 % among our population. Fig. 1 shows representative examples of 12 leads ECG of patients with pattern breaks in V2 and V3. Most patients presented as frequent PVC (89.4 %), and the remaining presented as VT.

### 3.1 Baseline characteristic

Table 1 displays the baseline characteristics of the patients. There is no difference between the two groups except for the diagnosis of diabetes mellitus (DM). There are more patients diagnosed with DM in Group 2 compared to Group 1 (28.6 % vs. 1.9 %, *P* = 0.006). The Body Mass Index (BMI) is insignificantly higher in Group 2 patients compared to Group 1 patients (25.1 ± 5.8 vs. 22.8 ± 4.1 kg/m^2^, *P* = 0.087). The majority of patients (49 patients, 76.6 %) exhibited normal left ventricular ejection fraction (LVEF), as evaluated by transthoracic echocardiography (TTE) and/or cardiac magnetic resonance imaging (CMR), with a median LVEF of 56.5 % (IQR 50.4 %–60.6 %). Specifically, only 6.1 % and 16.7 % of patients had an LVEF <40 % and 40–49 %, respectively. In patients with decreased LVEF, CMR demonstrated no late gadolinium enhancement.

**Table 1:** Baseline characteristics.

Characteristics (N = 66)		Group 1: Successful Ablation and no recurrence (N = 52)	Group 2: Failed ablation (N = 5) or VA recurrence (N = 9) (Total N = 14)	p-value
Age (in years ± SD)	42.3 ± 15.3	41.4 ± 13.7	45.3 ± 20.5	0.402
Males, N (%)	26 (39.4)	18 (34.6)	8 (57.1)	0.126
Body Mass Index in kg/m^2^	23.3 ± 4.3	22.8 ± 4.1	25.1 ± 5.8	0.087
Co-Morbidities, N (%)				
Hypertension	10 (15.2)	6 (11.5)	4 (28.6)	0.247
Diabetes Mellitus	5 (7.6)	1 (1.9)	4 (28.6)	0.006
Thyroid dysfunction	1 (1.5)	0 (0.0)	1 (7.1)	0.478
Dyslipidemia	8 (12.1)	6 (11.5)	2 (14.3)	1.000
Coronary artery disease	4 (6.1)	1 (1.9)	0 (0.0)	1.000
History of stroke	1 (1.5)	3 (5.8)	1 (7.1)	1.000
Prior history of ablation, N (%)	5 (7.6)	5 (9.6)	0 (0.0)	0.524
Prior VA ablation	4 (6.1)	4 (7.7)	0 (0.0)	0.660
VA symptoms, N (%)	61 (92.4)	49 (94.2)	12 (85.7)	0.617
Palpitation	51 (77.3)	41 (78.8)	10 (71.4)	0.819
Syncope or Presyncope	13 (19.7)	10 (19.2)	3 (21.4)	1.000
Dyspnea	12 (18.2)	8 (15.4)	4 (28.6)	0.456
Chest pain/ tightness/discomfort	28 (42.4)	21 (40.4)	7 (50.0)	0.518
Clinical Manifestation				
Frequent PVC	59 (89.4)	46 (88.5)	13 (92.9)	1.000
VT	7 (10.6)	6 (11.5)	1 (7.1)	
LVEF				
≥50 %	51 (77.3 %)	39 (79.6)	10 (71.4)	0.794
40 – 49 %	11 (16.7 %)	7 (14.3)	3 (21.4)	
<40 %	4 (6.1 %)	3 (6.1)	1 (7.1)	
PVC burden in %	18.8 ± 9.5	19.0 ± 9.6	18.0 ± 9.4	0.743

VA = ventricular arrhythmia; PVC = premature ventricular contraction; VT = ventricular tachycardia; LVEF = left ventricular ejection fraction; IQR = interquartile range.

### 3.2 ECG analysis

Most patients presented with PBV2 with a transitional zone at V3 or V4. The details of the V2S/V3R ratio, V2 transition ratio, and other parameters are described in Table 2 [7], [8].

**Table 2:** Comparison of Procedural parameters between two Groups.

ECG Parameters	Total (N = 66)	Group 1: Successful Ablation and no recurrence (N = 52)	Group 2: Failed ablation (N = 5) or VA recurrence (N = 9) (Total N = 14)	P Value*
VA characteristics				
Precordial transition				
V3	28 (42.4)	22 (42.3)	6 (42.9)	0.999
V4	38 (57.6)	30 (57.7)	8 (57.1)	
Break pattern				
V2	60 (90.9)	46 (88.5)	14 (100)	0.328
V3	6 (9.1)	6 (11.5)	0 (0)	
QRS duration (ms)	158.79 ± 14.00	159.52 ± 13.73	156.07 ± 15.18	0.418
CI (ms)	464.41 ± 75.62	465.85 ± 71.04	459.07 ± 93.57	0.769
I R amplitude (mV)	0.14 ± 0.23	0.16 ± 0.25	0.08 ± 0.16	0.258
I S amplitude (mV)	0.34 ± 0.37	0.34 ± 0.4	0.34 ± 0.21	0.958
II R amplitude (mV)	1.83 ± 0.53	1.81 ± 0.53	1.89 ± 0.56	0.656
III R amplitude (mV)	1.96 ± 0.78	1.95 ± 0.81	2.00 ± 0.70	0.811
II/III R amplitude ratio	1.06 ± 0.21	1.06 ± 0.22	1.06 ± 0.17	0.900
III/II R amplitude ratio	0.99 ± 0.22	0.99 ± 0.23	0.97 ± 0.19	0.720
aVR Q amplitude (mV)	0.88 ± 0.25	0.89 ± 0.25	0.85 ± 0.25	0.596
aVL Q amplitude (mV)	1.10 ± 0.58	1.09 ± 0.61	1.12 ± 0.47	0.855
aVR/aVL Q ratio	1.28 ± 0.57	1.25 ± 0.59	1.38 ± 0.53	0.461
aVF R amplitude (mV)	1.89 ± 0.66	1.88 ± 0.68	1.95 ± 0.60	0.715
V1 R amplitude (mV)	0.35 ± 0.18	0.35 ± 0.17	0.39 ± 0.19	0.391
V1 S amplitude (mV)	1.20 ± 0.49	1.22 ± 0.52	1.11 ± 0.40	0.482
V1 QRS amplitude (mV)	1.55 ± 0.53	1.56 ± 0.57	1.50 ± 0.37	0.715
V2 R amplitude (mV)	0.28 ± 0.20	0.28 ± 0.21	0.30 ± 0.18	0.686
V2 S amplitude (mV)	1.98 ± 0.73	1.98 ± 0.71	1.98 ± 0.82	0.966
V2 QRS amplitude (mV)	2.26 ± 0.73	2.25 ± 0.71	2.28 ± 0.80	0.878
V2 R/S ratio	0.18 ± 0.22	0.18 ± 0.24	0.18 ± 0.14	0.971
V2 R/QRS ratio	0.13 ± 0.10	0.13 ± 0.10	0.14 ± 0.09	0.741
V2/V1 R ratio	0.81 ± 0.54	0.83 ± 0.60	0.73 ± 0.13	0.539
V2/V3 R ratio	0.51 ± 0.34	0.52 ± 0.37	0.50 ± 0.24	0.830
V3 R amplitude (mV)	0.63 ± 0.37	0.61 ± 0.37	0.69 ± 0.39	0.459
V3 S amplitude (mV)	0.87 ± 0.84	0.86 ± 0.81	0.93 ± 0.95	0.795
V3 QRS amplitude (mV)	1.50 ± 0.74	1.47 ± 0.72	1.62 ± 0.81	0.504
V2S/V3R ratio	4.72 ± 3.95	4.91 ± 4.16	4.01 ± 3.06	0.455
V2 transition ratio	0.52 ± 0.50	0.51 ± 0.45	0.57 ± 0.68	0.715
Transitional zone index (TZI)	0.303 ± 1.07	0.29 ± 0.95	0.36 ± 1.47	0.833
Mapping strategy				
LAT or pace mapping only, N (%)	24 (36.4)	18 (34.6)	6 (42.9)	0.569
LAT and pace mapping, N (%)	42 (63.6)	34 (65.4)	8 (57.1)	
Effective ablation site (suppression or success)				
RVOT	57 (86.4)	46 (88.5)	11 (78.6)	0.916
LVOT	5 (7.6)	2 (3.8)	3 (21.4)	0.027
Multiple sites	4 (6.1)	4 (7.7)	0 (0)	0.571
Distance from commissure to RVOT (mm)	3.26 ± 0.74	3.04 ± 0.58	4.10 ± 0.68	<0.001

CI = coupling interval.

* Comparison between Group 1 and Group 2.

### 3.3 Mapping and ablation

All patients underwent a comprehensive three-dimensional electroanatomical mapping procedure. Local activation time (LAT) mapping and/or pace mapping were conducted for all individuals. LAT mapping was performed in 54 (81.8 %) patients, with the average earliest LAT (before the onset of QRS) identified during mapping being 36.1 ± 7.6 ms, predominantly observed in the RVOT in 89.5 % of cases, notably in the anterior septal aspect of the RVOT (56.1 %), with lesser occurrences in the posterior septal aspect of RVOT and mid-septal aspect of RVOT (22.8 % and 10.5 %, respectively). Pace mapping was performed in 52 (78.8 %) patients and revealed a high correlation with a median of 97.0 % with clinical PVC. Moreover, the most optimal pace map location was consistently identified within the RVOT (96.1 %), primarily in the anterior septal region (59.6 %), followed by the posterior septal RVOT and mid- septal RVOT (25.0 % and 11.5 %, respectively).

**Fig. 3:**
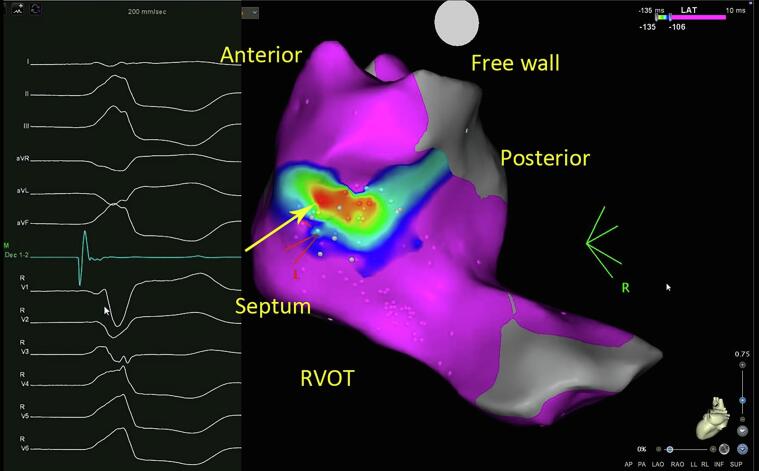
Example of successful site ablation of VA with pattern break at V2 was at anterior RVOT**.** Abbreviation: VA, ventricular arrhythmia; RVOT, right ventricular outflow tract.

Acute procedural success was achieved in 61 patients (92.4 %). Table 3 shows the final successful ablation site. Similar to the LAT and pace mapping, the final successful ablation site most commonly (88.5 %) from RVOT (Fig. 3), specifically from anterior septal RVOT, posterior septal RVOT, and mid septal RVOT in descending order.

**Table 3:** Successful ablation site.

Successful site of ablation (N = 61)	No. (%)
RVOT	54 (88.5)
Anterior septum	34 (55.7)
Posterior septum	15 (24.6)
Mid septum	5 (8,2)
LVOT	3 (4.9)
RCC	1 (1.6)
LCC	0 (0)
RCC/LCC junction	2 (3.3)
Multiple sites	4 (6.6)

RVOT = right ventricular outflow tract; LVOT = left ventricular outflow tract; RCC = right coronary cusp; LCC = left coronary cusp; GCV = great cardiac vein

In three patients (4.9 %), the final successful ablation site was from LVOT, specifically two patients from the commissure between LCC/RCC and one from RCC close to the commissure. Two patients needed ablation from both the supravalvular and subvalvular of the LCC/RCC commissure, and one patient only needed ablation from the subvalvular area. The VA needed ablation from multiple sites in four patients (6.6 %). Among patients who needed ablation from multiple sites, the effective ablation location was from anterior septal and mid-septal RVOT; anterior septal RVOT and LCC/RCC commissure; anterior septal RVOT and LCC/RCC commissure; and posterior septal RVOT, and LCC/RCC commissure.

The overall acute success rate among six patients with PBV3 was 100 %. The successful ablation site was anterior septal RVOT in four patients, posterior septal RVOT in one patient, and multiple sites (anterior septal RVOT and LCC/RCC commissure) in one patient.

Five patients failed to achieve acute ablation success. The earliest LAT and best pace map of those patients were two patients from posterior septal RVOT, two from LCC/RCC commissure, and one from anterior septal RVOT.

**Fig. 4:**
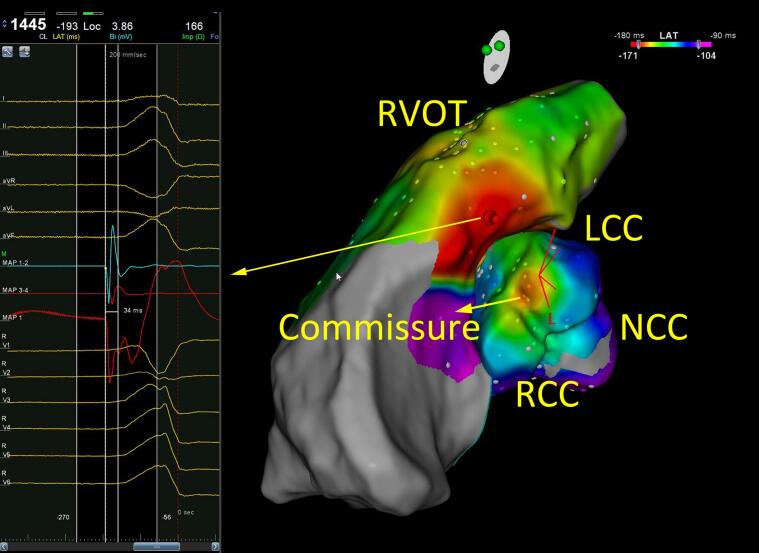
Example of successful site ablation of VA with pattern break at V2 was at anterior RVOT with LVOT mapping, which revealed the earliest activation site in LCC/RCC commissure. Abbreviation: LVOT, left ventricular outflow tract; LCC, left coronary cusp; RCC, right coronary cusp VA, ventricular arrhythmia; RVOT, right ventricular outflow tract.

Among the 57 patients who received RVOT-only ablation, ten patients received LVOT mapping before the energy delivery. In these ten patients, the earliest activation site was LCC/RCC commissure (Fig. 4).

### 3.4 Anatomic distance between LCC/RCC commissure and RVOT

The image of the CMR was exported for offline analysis using Horos software. The image quality and format were available for analysis in 56 patients. The mean distance from LCC/RCC commissure to RVOT (DCR) was 2.90 ± 0.99 mm. The DCR in Group 1 is significantly shorter than Group 2 (2.67 ± 0.70 vs. 4.37 ± 1.43, p = 0.001).

### 3.5 Follow up

The median follow-up period was 37 months. Nine patients (15.0 %) had recurrence during follow-up. All recurrences occurred with similar PVC morphology during the first year of follow-up, with a median of two months (range 1 – 8 months). This overall long-term success rate is 78.8 %. Five recurrent cases underwent redo ablation, in which four patients achieved acute procedural success with the ablation at a similar location to the previous procedure. No recurrence was found for at least one year follow-up after the repeat procedure.

Several clinical parameters were analyzed to predict the composite endpoint of acute procedural failure and recurrence (Table 4). After multivariate analysis, the diagnosis of DM (OR: 39.13, 95 % CI: 1.31–1173.49, p = 0.035) and the distance from LCC/RCC commissure to RVOT (OR: 18.86, 95 % CI: 3.38–105.17, p = 0.001) independently associated with the procedural failure or recurrence.

**Table 4:** Univariate & Multivariate Analysis for the Long-Term Recurrence or Failure.

**Variables**	Univariate			Multivariate		
Parameters	P value	Odds Ratio	95 % CI	P value	Odds Ratio	95 % CI
Age, (year)	0.397	1.02	0.98–1.06	—	—	—
Men	0.132	2.52	0.76–8.39	—	—	—
Body Mass Index in kg/m^2^	0.097	1.11	0.98–1.26	0.859	0.98	0.82–1.18
Diabetes mellitus	0.010	20.4	2.06–202.2	0.035	39.13	1.31–1173.49
Effective in LVOT	0.059	6.27	0.93–42.2	0.289	3.92	0.31–49.02
Distance from commissure to RVOT (mm)	<0.001	16.28	3.75–70.65	0.001	18.86	3.38–105.17

Values are number of events (%) unless otherwise indicated.

RVOT, right ventricular outflow tract; SD, standard deviation.

There were no significant ST-T changes and no vascular event in the follow-up period after the procedure.

## 4 Discussion

### 4.1 Main findings

The current study provided detailed clinical characteristics and ablation outcomes for Asian patients with outflow tract ventricular arrhythmias (VA) exhibiting a pattern break in precordial leads. The main findings were as follows: (1) The acute and long-term outcomes of these patients may not be as poor as those reported in Western countries; (2) The most common successful ablation site was the anterior septal right ventricular outflow tract (RVOT) in VA with a break pattern; (3) The history of DM and a long distance from LCC/RCC commissure to RVOT was associated with a failed procedure or recurrence after multiple attempts. (4) A new subset of patients with a unique pattern break in V3, who exhibited excellent prognosis with final ablation success site primarily in the RVOT.

### 4.2 Ablation outcomes

Our data showed that the most common earliest LAT and best pace mapping site was from RVOT, specifically from anterior septal RVOT. This is consistent with the previous study by Hayashi et al. [4]. Our data also showed that the most frequent successful ablation site was from RVOT (92.4 %), with anterior septal RVOT (55.7 %) being the most common successful ablation site.

It was hypothesized that PVC with PBV2 usually originated near the interventricular groove where proximal LAD courses. The proximity of RVOT and proximal LAD has been described before, and ablation in the RVOT region, most commonly in anterior RVOT, might cause LAD injury in several previous case reports [9], [10], [11]. The proximity of the PVC origin to the LAD may explain the difficulties and low success rate in the previous cohort [4]. However, we could not confirm this in our cohort, as we did not perform coronary angiography routinely in PVC cases.

Our data for acute and late success rates after a median follow-up of 37 months were 92.4 % and 78.8 %. These are higher than the previous report (58.3 % and 41.7 %, respectively) [4]. The exact cause of this difference is still unclear. The previous study was conducted in the Western population. We hypothesized that the better outcome in the Asian population was related to the anthropometric differences between the two populations. The Asian population has a smaller heart and body size [12], [13], [14], [15]. Our cohort only has a median BMI of 22.0 kg/m^2^, which might correspond with a smaller cardiac mass. The smaller cardiac mass may make it easier for radiofrequency energy during ablation to penetrate the interventricular groove, translating into better clinical outcomes.

Previous reports have described the differences in several ECG parameters between Asians and Whites [16], [17], [18]. However, no study has been published specifically comparing the ECG differences in patients with outflow tract VA between Asian and Western populations.

In our cohort, the earliest activation site in the LVOT was mostly localized at the LCC/RCC commissure, regardless of the successful ablation site. These findings suggest a possible intramural origin of the VA. The observed association between the long distance from the LCC/RCC commissure to the RVOT and procedural failure further supports the hypothesis of an intramural focus within this area.

In a small proportion of patients (6.6 %), the VA needed ablation from multiple sites, also suggesting an intramural origin of the VA. Multiple-site ablation was one hundred percent successful, and there was no recurrence during follow-up. It is also interesting to note that all successful multiple-sites ablation also involved ablation from RVOT. The higher percentage of failed ablations and VA recurrences of patients with ablation only at LVOT may partly support how important ablation in the RVOT area is. A recent randomized controlled trial by Wang and colleagues confirmed that additional ablation surrounding the target point might improve ablation outcomes compared with the single-point ablation strategy [19]. Due to the probable intramural origin of the PVC, ablation energy might need to target the surrounding area from another cardiac chamber.

Limited studies have examined catheter ablation outcomes in diabetic patients. While ablation effectively reduces PVC burden, diabetic patients show less LV function recovery and have lower long-term arrhythmia-free survival [20], [21], [22], a trend also observed in our study.

### 4.3 Pattern break in precordial V3

We also described a small subset of patients with PBV3. The prevalence of this subset is very low, with only 9.1 % of patients with pattern break in precordial lead and 0.6 % of our patients’ population. To the best of our knowledge, this is the first study reporting this rare subset of patients. The underlying mechanism responsible for this ECG finding remains undetermined. Ventricular arrhythmia originating from anterior and posterior interventricular septum are associated with pattern break in V2 and its opposite ECG features, respectively [4], [23]. It is logical to assume that PBV3 is also associated with anterior interventricular groove location, probably in patients with slight heart rotation or shift. The prognosis of this patient subset was excellent, with a 100 % acute and late success rate. All patients with PBV3 needed ablation in RVOT to achieve successful ablation.

### 4.4 Anatomic location

When reviewing the anatomical location of the aortic cusp, coronary artery, and RVOT, it was observed that the pulmonary cusp is located superiorly and anteriorly to the coronary cusp. The left anterior descending (LAD) artery passes along the lateral side of the upper left coronary cusp (LCC) and the junction between the anterior RVOT and the pulmonary cusp. In our study cohort, the commissure of the LCC/RCC appeared lower than the pulmonary cusp and faced the septal side of RVOT (Fig. 2).

In cases requiring bilateral mapping (N = 22), the earliest activation in the left ventricular outflow tract (LVOT) was mainly localized at the commissure of the LCC/RCC (95.5 %, 21/22, Fig. 4). The great cardiac vein mapping in our study cohort could not identify earlier activation signals compared to the endocardial mapping, which might exclude the origin as the septal summit [24]. In reviewing the case with PB, the junction between the commissure of the RCC/LCC and the anterior RVOT septum is below the LAD. The lower extension of this junction is connected to the upper interventricular septum. Due to its proximity to the LV summit and anatomical distinction from the LV summit and septal summit, we refer to this area as the “inner summit.” Preferential conduction between the RVOT and LVOT was observed in previous publications [25]. The origin of VA from this area might exhibit preferential conduction, resulting in PB on the surface ECG.

The ECG characteristics are different from the previous publication describing the VA from LCC/RCC aortic cusp with a QS morphology in lead V1 with notching on the downward deflection with precordial transition at lead V3 [26]. The difference could be attributed to the deep-seated or intramural origin close to the RVOT area in our study population. We could observe a low success rate in the patients with relatively earlier activation in the LVOT compared to RVOT. This hypothesis is consistent with our finding that the distance between LCC/RCC to RVOT is independently associated with procedural failure or recurrence.

### 4.5 Study limitation

The study has several limitations that warrant consideration. First, the retrospective design may introduce some information bias. The reliance on patient self-reporting and external medical records for follow-up data could introduce reporting biases. Second, the study was conducted at two institutions, both in East Asia. This may limit the generalizability of the findings to other Asian populations. However, previous studies consistently showed the smaller anthropometric parameters of different regions of the Asian population compared with the Western population [13]. Third, the absence of routine coronary angiography in our cohort means that the proximity of the successful ablation site to coronary arteries was unknown. Fourth, we did not perform systematic mapping in both RVOT and LVOT in all patients. The endocardial activation maps may be misleading when the PVCs are intramural. We rarely use high-density mapping catheters for mapping and did not map the septal perforators with wire or micro catheters to exclude the septal summit as the site of arrhythmia origin. Last, we indirectly compared our acute success and late success rate with the previous study. A study with a direct head-to-head outcome would indeed be more ideal.

## 5 Conclusion

Outflow tract ventricular arrhythmia with a pattern break at precordial lead in the Asian population may not have a worse clinical outcome than previously reported in Western countries. The most common acute success site for ablation was RVOT, specifically anterior RVOT. A long anatomic distance from the LCC/RCC commissure to the RVOT and DM diagnosis was associated with an unsatisfied ablation outcome.

## 6 Author statement

Muhammad Rafdi Amadis: This author takes responsibility for all aspects of the reliability and freedom from bias of the data presented and their discussed interpretation.

Satoshi Higa: This author takes responsibility for all aspects of the reliability and freedom from bias of the data presented and their discussed interpretation.

Chin-Yu Lin: This author takes responsibility for all aspects of the reliability and freedom from bias of the data presented and their discussed interpretation.

Yuen Hoong Phang: This author takes responsibility for all aspects of the reliability and freedom from bias of the data presented and their discussed interpretation.

Chia-Hsin Chiang: This author takes responsibility for all aspects of the reliability and freedom from bias of the data presented and their discussed interpretation.

Jose Antonio Lopez Bautista^:^ This author takes responsibility for all aspects of the reliability and freedom from bias of the data presented and their discussed interpretation.

Yenn-Jiang Lin: This author takes responsibility for all aspects of the reliability and freedom from bias of the data presented and their discussed interpretation.

Shih-Lin Chang: This author takes responsibility for all aspects of the reliability and freedom from bias of the data presented and their discussed interpretation.

Li-Wei Lo: This author takes responsibility for all aspects of the reliability and freedom from bias of the data presented and their discussed interpretation.

Yu-Feng Hu: This author takes responsibility for all aspects of the reliability and freedom from bias of the data presented and their discussed interpretation.

Fa-Po Chung: This author takes responsibility for all aspects of the reliability and freedom from bias of the data presented and their discussed interpretation.

Ting-Yung Chang: This author takes responsibility for all aspects of the reliability and freedom from bias of the data presented and their discussed interpretation.

Ling Kuo: This author takes responsibility for all aspects of the reliability and freedom from bias of the data presented and their discussed interpretation.

Cheng-I Wu: This author takes responsibility for all aspects of the reliability and freedom from bias of the data presented and their discussed interpretation.

Chih-Min Liu: This author takes responsibility for all aspects of the reliability and freedom from bias of the data presented and their discussed interpretation.

Shin-Huei Liu: This author takes responsibility for all aspects of the reliability and freedom from bias of the data presented and their discussed interpretation.

Ming-Jen Kuo: This author takes responsibility for all aspects of the reliability and freedom from bias of the data presented and their discussed interpretation.

Thien-Chuong Nguyen-Khac: This author takes responsibility for all aspects of the reliability and freedom from bias of the data presented and their discussed interpretation.

Guan-Yi Li: This author takes responsibility for all aspects of the reliability and freedom from bias of the data presented and their discussed interpretation.

Yu-Shan Huang: This author takes responsibility for all aspects of the reliability and freedom from bias of the data presented and their discussed interpretation.

Shih-Ann Chen: This author takes responsibility for all aspects of the reliability and freedom from bias of the data presented and their discussed interpretation.

## Acknowledgement of grant support

This work was supported by the Biosense Webster IIS (C2304900), Ministry of Science and Technology (NSTC 113 - 2314 - B - 075 - 029 - MY3, NSTC 113 - 2628 - B - 075 - 003 - MY3, MOST 111 - 2314-B-075 - 007-MY3); and Taipei Veterans General Hospital (grant no. V113C-044, C19-027), Veterans General Hospitals and University System of Taiwan Joint Research Program Joint Research Program (VGHUST114-G1-4-1).

## CRediT authorship contribution statement

**Muhammad Rafdi Amadis:** Writing – original draft, Visualization, Software, Methodology, Investigation, Formal analysis, Data curation, Conceptualization. **Satoshi Higa:** Writing – review & editing, Visualization, Validation, Supervision, Project administration, Data curation, Conceptualization. **Chin-Yu Lin:** Writing – review & editing, Visualization, Validation, Supervision, Resources, Project administration, Methodology, Investigation, Funding acquisition, Formal analysis, Conceptualization. **Yuen Hoong Phang:** Writing – original draft, Investigation, Formal analysis, Data curation. **Chia-Hsin Chiang:** Methodology, Investigation, Formal analysis. **Jose Antonio Lopez Bautista:** Data curation, Conceptualization. **Yenn-Jiang Lin:** Writing – review & editing, Validation, Supervision. **Shih-Lin Chang:** Writing – review & editing, Validation, Supervision. **Li-Wei Lo:** Writing – review & editing, Validation, Supervision. **Yu-Feng Hu:** Writing – review & editing, Validation, Supervision. **Fa-Po Chung:** Writing – review & editing, Validation, Supervision, Conceptualization. **Ting-Yung Chang:** Writing – review & editing, Validation, Supervision, Conceptualization. **Ling Kuo:** Writing – review & editing, Validation, Supervision. **Cheng-I Wu:** Writing – review & editing, Validation, Supervision. **Chih-Min Liu:** Writing – review & editing, Validation, Supervision. **Shin-Huei Liu:** Writing – review & editing, Validation, Supervision. **Ming-Jen Kuo:** Writing – review & editing, Validation, Supervision. **Thien-Chuong Nguyen-Khac:** Writing – review & editing. **Guan-Yi Li:** Writing – review & editing, Validation, Supervision. **Yu-Shan Huang:** Writing – review & editing, Validation, Supervision. **Shih-Ann Chen:** Writing – review & editing, Validation, Supervision.

## Declaration of competing interest

The authors declare the following financial interests/personal relationships which may be considered as potential competing interests: Chin-Yu Lin reports financial support was provided by Biosense Webster IIS. Chin-Yu Lin reports financial support was provided by Ministry of Science and Technology. If there are other authors, they declare that they have no known competing financial interests or personal relationships that could have appeared to influence the work reported in this paper.
