# Supplementary figures and images for: Reappraisal of the clinical and anatomic characteristics of idiopathic outflow tract ventricular arrhythmias with an R wave pattern break in precordial lead: A multi-center study
Source: Int J Cardiol Heart Vasc. 2025 Mar 25;58:101664. doi: 10.1016/j.ijcha.2025.101664 (PMC11985138; doi:10.1016/j.ijcha.2025.101664)

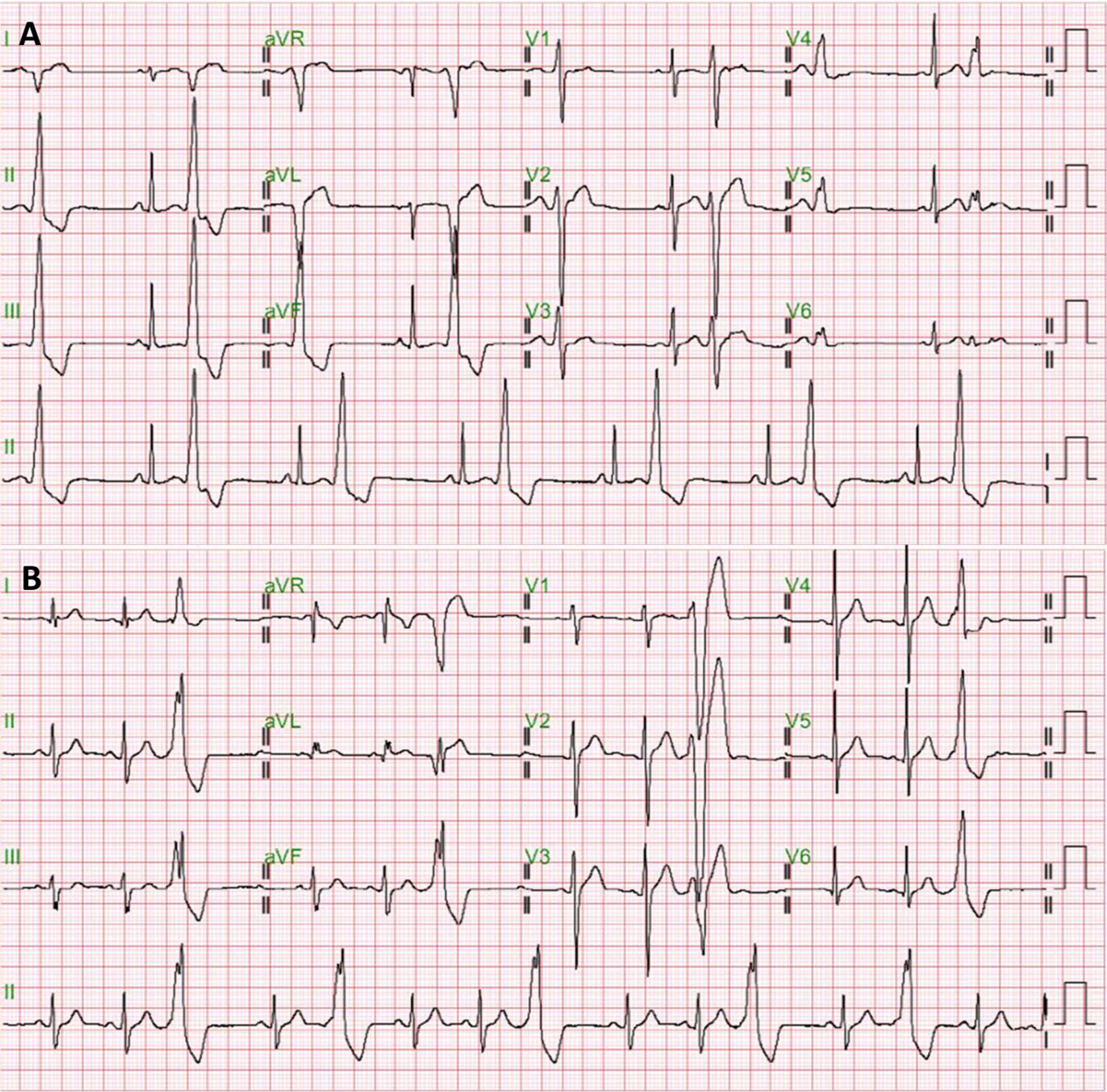

Supplement: Supplementary Fig. 1 [file mmc1.jpg]
